# Big Data and the Brain: Peeking at the Future

**DOI:** 10.1016/j.gpb.2019.11.003

**Published:** 2019-12-04

**Authors:** Hongzhu Qu, Hongxing Lei, Xiangdong Fang

**Affiliations:** 1 CAS Key Laboratory of Genome Sciences and Information, Beijing Institute of Genomics, Chinese Academy of Sciences, Beijing 100101, China; 2 Sino-Danish College, University of Chinese Academy of Sciences, Beijing 100049, China; 3 Institute for Stem Cell and Regeneration, Chinese Academy of Sciences, Beijing 100101, China; 4 College of Life Sciences, University of Chinese Academy of Sciences, Beijing 100049, China

## The era of brain science across the world

The human brain is the most complex organ in the human body. It comprises billions of neurons and supporting cells in a complex network, managing everything from physical functions to thoughts and feelings of humans. Dysfunctions of the complex network caused by both genetic and environmental factors result in many brain disorders. Several large brain projects have been launched, aiming to understand how the brain works in health and disease, including the Human Brain Project of the European Union, the Brain Research through Advancing Innovative Neurotechnologies (BRAIN) Initiative of the United States, and the Brain Mapping by Integrated Neurotechnologies for Disease Studies (Brain/MIND) project of Japan. These projects gather, organize, and disseminate data describing the brain and related diseases, in the meantime develop brain-inspired computing and data analytics. As an example, the Human Connectome project (HCP) uses cutting-edge brain imaging technologies to map the circuitry of the healthy adult human brain in order to track their susceptibility to change during aging and disorder occurrence. In addition, the HCP will collect DNA samples, demographic information and behavioral data from the subject to investigate the effect on human brain by both genetics and environment. By analyzing the functional magnetic resonance imaging (fMRI) mappings to detect the factors determining face recognition, the code for facial identity comprised of 205 neurons in primate brain was discovered [1], which reveals how brain recognize the faces of people and shines a light on the research in brain science by artificial intelligent.

China Brain Project (CBP) has recently been initiated to promote major advances in the basic understanding of brain and address some urgent societal needs on human health [2]. CBP puts the research on brain disorders and brain-inspired artificial intelligence as immediate high-priority areas (so called “one body with two wings”). More importantly, the large macaque monkey resources and the advancement in developing human disease models using macaque monkey in China provide the opportunity to study higher cognitive functions of brain and pathogenic mechanisms of brain disorders [2]. To understand how the brain works, we need to identify the diverse types of cells (neurons and glia) and their distributions, the dynamic molecular expression patterns in each cell type, and the network among molecular markers in different types of cells at different states of the brain. Therefore, three types of maps, cell type map, connectivity map, and activity map, need to be built [2]. Among them, ‘connectivity map’, the neuron network among all cell types at the spatial resolution of single neurons, is different from the connectome in HCP, which is the brain circuitry at a rather low spatial resolution by MRI technology. ‘Cell type map’ will be depicted in an increasing rate with the development of single-cell sequencing technologies.

## Single-cell sequencing technology boosts the study of brain science

Technologies for single-cell amplification and sequencing are maturing, which will be applied by more researchers as a routine tool to assess the genome, transcriptome, proteome, and epigenome of single cells to gain biological insights, particularly in brain science. To date, single-cell transcriptome is the most widely used single-cell sequencing technology, especially in model organism mice [3], [4], [5] or zebrafish [6], for studying the mechanisms of brain development and the pathogenesis of brain related diseases. Currently, it becomes a routine tool for brain study directly in human specimens. Zhong et al*.*, for the first time, systematically explored the cellular and molecular mechanisms involved in the formation of prefrontal lobes in the development of the human prefrontal cortex (PFC) by single-cell RNA sequencing (scRNA-seq) [7]. They identified 35 subtypes of cells in six main classes from the developing PFC and traced the developmental trajectories of these cells. The diverse neuron subtypes likely contribute to the cellular basis of the elaborate circuit formation in human brains. The identification of new intermediate progenitor cell markers and their biological mechanisms is helpful for investigating the molecular basis of neurological disorders and social cognition deficits. Other types of cells from different regions of the human brain, such as microglia [8], midbrain dopamine (mDA) neurons [9], brain organoids [10], were recently used to characterize their cell subgroups and expression profiles, and to identify new targets for the treatment of neurodegenerative and neuroinflammatory pathologies. Besides the cell type identification in brain, how individual neurons in any region of neocortex convey information to their targets is also a concern, which to some extent belongs to the activity map. Using whole-brain fluorescence-based axonal tracing and high-throughput DNA sequencing of genetically barcoded neurons (MAPseq), signals carried by individual cortical neurons are observed to be shared across subsets of target areas in mouse primary visual cortex [11]. This divergent nature of information transmission may therefore help to construct models of hierarchical sensory processing.

The underlying causes of neurological and psychiatric disorders, such as Alzheimer’s disease (AD), Parkinson’s disease (PD), and major depression, are as important as the principles of how the brain works in normal state. Genome-wide association study (GWAS) has been the primary method to explore the genetic risk of common genetic diseases, like PD. Recently the subpopulations of dopaminergic neurons that are linked to PD in the mouse brain at embryonic and early postnatal time points were characterized by scRNA-seq [12]. The expression levels of population-specific genes were used to develop a scoring system to prioritize sporadic PD-risk genes [12], providing biologically pertinent candidates and a new approach for genetic research of PD in humans. Additionally, an unexpected tetrasomy 1q was found in ∼20% of neurons by single-cell whole-genome sequencing on a patient with hemimegalencephaly due to a somatic copy number variation (CNV) of chromosome 1q [13]. Malignant tumors, as one of the main diseases influencing human health, have attracted scientists’ attention for their rapid increasing morbidity. Single-cell sequencing technology has been applied to the study of tumor heterogeneity, subpopulations of proliferating stem-like cells, and the tumor microenvironment of glioma, the most common primary central nervous system tumor in adults [14], [15], [16]. The consistent gene signature shared among infiltrating glioblastoma cells [17], genetic alternations in different cell types, and the composition of tumor microenvironment within glioma may facilitate the potential diagnosis of such disease.

## Integrative analysis of big data is a path forward in brain science

Brain science has entered a new era of big data with the development of single-cell sequencing technologies, the development of new tools for mapping neuronal connections, the increasing resolution of imaging technologies, and the explosion of nanoscience. Many large brain projects and individual studies have accumulated a huge amount of data related to brain function. Taking the HCP as an example, it released high quality image data from 1100 subjects plus out-of-scanner, behavior-only data from 100 subjects, which have an unprecedented spatial resolution of 1.25 mm and very low structured temporal noise [18]. The first living human cells database created by the Allen Institute includes gene expression data of 16,000 individual cells from only 3 people, and the electrical properties of 300 different types of neuron from 36 patients, along with 3-D reconstructions of the spidery shapes of some of them, and computer models that simulate their electrical behavior so far [19], these data are from small pieces of brain that neurosurgeons have discarded during surgery with the consent of patients The future projects of large cohort with brain disorders will provide unpredictable amount of omics data including genomics, transcriptomics, epigenomics, proteomics, and metabolomics. At the same time, a myriad of medical information about the brain disorders will also be integrated into the huge pool to illuminate how the brain works normally and how it might be changed during diseases. These massive datasets have provided us the unprecedented opportunity to study the function of human brains comprehensively.

We are facing a real big challenge with the increasingly complex datasets in brain science. First, for each type of data, there is a lack of standard specification for their generation, storage, processing, and utilization, which hampers the comparison and integration of data or results from different research groups. Moreover, most of the data are unstructured, heterogeneous, and generated from different sources. Developing efficient tools to transform them into structured and normalized format is of critical importance. Second, how to integrate all different types of data including medical information, omics data, morphology, and connectivity images to gain a deep understanding of the brain’s role is a bottleneck. In addition, the technologies of the internet, artificial intelligence, augmented reality (AR), and virtual reality (VR) will be gradually applied to the study of brain science. Improved statistical models are needed for reliable inference from these data. Computational strategies are becoming central to virtually everything we do now. Third, storage and processing of such large datasets with high efficiency remain difficult. The rapid rate of data generation requires powerful storage and processing ability. New processing and computing methods as well as storage devices are needed to bridge the increasing gap between data growth and processing power. Advances in neuroimaging, genomics, computational neuroscience, and engineering have ushered us to another great era of brain science, when we are bound to make extraordinary discoveries regarding normal brain activity, disorders of the brain, and our very sense of self.

In the era of big data, brain science is a fast-developing field. This special issue provides a glimpse of advances in the field of brain science, mainly focused on genomics and neuroimaging.

Genomics is at present one of the main workhorses for brain science. We specifically highlight the application of genomics tools to the investigation of brain disorders, including causal mutations, diagnostic biomarkers, and actionable targets. Built upon previous high-throughput sequencing studies, Ludwig et al. derived a model based on microRNAs to detect AD using peripheral blood [20]. Lin et al. constructed a knowledge base for *de novo* mutations in developmental disorders with special emphasis on isoform-specific mutations [21]. Yang et al. constructed a web server for the display and integration of multi-omics data for glioma [22]. Li J et al. summarized resources for brain transcriptome atlas and relevant computational pipelines [23]. Mu et al. reviewed progresses in single-cell genomics in connection to brain development and disorders [24]. Shen et al. summarized the diverse types of big data in PD, which pose great challenge for data integration and derivation of clinically-actionable models for disease prevention and management [25].

In the meantime, neuroimaging is increasingly utilized in the investigation of brain function and dysfunction. Liu et al. investigated cognitive dysfunction in type 2 diabetes using functional neural network [26]. Li X et al. reviewed three major challenges in the field of functional imaging and emphasized the need for better data integration strategies [27]. Wang et al. summarized the resources available for the investigation of neuropsychiatric disorders [28]. Li A et al. illustrated the challenges in processing the ever-increasing brainsmatic data and suggested some possible solutions [29]. Chen et al. emphasized the role of high-performance computing and deep-learning in deciphering the big data in brain science [30].

Through these snapshots, we hope the readers can achieve better understanding of the current status in brain science, especially the challenges we are facing and the road ahead of us.

## Competing interests

The authors have declared no competing interests.
